# Contaminations contaminate common databases

**DOI:** 10.1111/1755-0998.13272

**Published:** 2020-10-31

**Authors:** Staffan Bensch, Mizue Inumaru, Yukita Sato, Larisa Lee Cruz, Andrew A. Cunningham, Simon J. Goodman, Iris I. Levin, Patricia G. Parker, Patricia Casanueva, Maria‐Angeles Hernández, Gregorio Moreno‐Rueda, Maria‐Angeles Rojo

**Affiliations:** ^1^ Department of Biology MEEL Lund University Lund Sweden; ^2^ Laboratory of Biomedical Science Department of Veterinary Medicine College of Bioresource Sciences Nihon University Fujisawa Japan; ^3^ Maison de la Télédétection Montpellier France; ^4^ Institute of Zoology Zoological Society of London London UK; ^5^ School of Biology Faculty of Biological Sciences University of Leeds Leeds UK; ^6^ Department of Biology Kenyon College Gambier OH USA; ^7^ Department of Biology and Whitney R. Harris World Ecology Center University of Missouri –St. Louis St. Louis MO USA; ^8^ Departamento de Ciencias Experimentales Universidad Europea Miguel de Cervantes, Calle Padre Julio Chevalier Valladolid Spain; ^9^ Departamento de Biología Ambiental Facultad de Ciencias Universidad de Navarra Pamplona Spain; ^10^ Departamento de Zoología Universidad de Granada Granada Spain

**Keywords:** *Haemoproteus*, haemosporidian parasites, PCR contamination, plasmodium, sequence databases

## Abstract

The polymerase chain reaction (PCR) is a very powerful method to detect and identify pathogens. The high sensitivity of the method, however, comes with a cost; any of the millions of artificial DNA copies generated by PCR can serve as a template in a following experiment. If not identified as contaminations, these may result in erroneous conclusions on the occurrence of the pathogen, thereby inflating estimates of host range and geographic distribution. In the present paper, we evaluate whether several published records of avian haemosporidian parasites, in either unusual host species or geographical regions, might stem from PCR contaminations rather than novel biological findings. The detailed descriptions of these cases are shedding light upon the steps in the work process that might lead to PCR contaminations. By increasing the awareness of this problem, it will aid in developing procedures that keep these to a minimum. The examples in the present paper are from haemosporidians of birds, however the problem of contaminations and suggested actions should apply generally to all kinds of PCR‐based identifications, not just of parasites and pathogens.

## 1 INTRODUCTION

The invention and applications of polymerase chain reaction (PCR) and DNA sequencing have revolutionized research on wild organisms, including parasites and other pathogens. A few of the services provided by these methods include the unambiguous identification of morphologically difficult species, discoveries of cryptic species, delimitation of parasite host range, and resolution of life cycles. Furthermore, the generated DNA sequences can be used for constructing phylogenetic relationships. The importance of these contributions to the ecology and evolution of parasitic organisms cannot be overestimated. To facilitate data sharing it has become standard to report findings of parasites and pathogens based on DNA sequences in open DNA databases such as NCBI, but also in specialist databases delimited on various taxonomic levels or the organisms they infect, such as EuPathDB (Aurrecoechea et al., 2017) and PubMLST (Jolley et al., 2018).

The database MalAvi (http://130.235.244.92/Malavi/index.html) was initiated 10 years ago (Bensch et al., 2009) in order to structure the growing numbers of PCR‐based findings of bird blood parasites known as haemosporidians that include malaria parasites. The database presently contains >3,900 unique mitochondrial lineages (based on 478 bp of the cyt b gene) recorded from >1,800 species of birds and 58 species of blood‐sucking dipterans. It has become an important resource for analyses of the geographic distribution and host range of these globally distributed parasites (Rivero & Gandon, 2018), with 436 citations (web of science 2020‐04‐30) and steady annual growth of records. Clearly, the value of a database depends on the quality of the data. Incorrect records may inflate estimates of host species range and geographic distribution of the parasites. In this respect, false positives are more impactful than false negatives. Curated databases such as MalAvi are not resistant to erroneous records. However, as the knowledge of the typical distribution of parasites in host species or in geography are accumulating, unexpected findings can alert us to carefully check and confirm these before publication, and hence keep the database resources as accurate as possible.

In the present paper, we evaluate several unexpected findings of haemosporidian parasites in unusual host species or geographical regions that appear to be the results of PCR contamination or mix up of samples. We would like to emphasize that the authors of these papers are no guiltier of publishing records resulting from contamination than other researchers in the field, as the examples highlighted here probably represent only the tip of the iceberg of a widespread problem. It is only among parasites with restricted and known host and geographic distribution where we have a chance to detect potential cases of contaminations by using this broad approach. For most of the parasites, we do not know where and in what species a finding would be surprising, and hence, any errors among these will pass unnoticed. We believe that the detailed descriptions of these highlighted cases will elucidate what steps might lead to erroneous records and aid in developing procedures that keep these to a minimum. Although the examples in this paper all come from haemosporidians of birds, the problem of contaminations and suggested actions should apply generally to all kinds of PCR‐based identifications of parasites and pathogens and, indeed, of other organisms.

## 2 CASE STUDIES

In a study with the main focus of describing the species *Haemoproteus iwa* of Pelicaniform birds from the Galapagos (Levin et al., 2011), the authors additionally published sequences of three lineages obtained from blue‐footed boobies *Sula nebouxii*, also from the Galapagos. Phylogenetically, these were placed together with lineages of *Parahaemoproteus* of passerine birds, which was somewhat surprising, but as these records were not the focus of the study, the findings were not further discussed in the publication. At the time when the records were added to MalAvi, one of the lineages was shown to have a 100% match to a sequence (EMCIR1) obtained from a cirl bunting *Emberiza cirlus* in Bulgaria (Dimitrov et al., 2010). This was indeed very surprising, as both the locations (12,400 km apart) and the supposed host species (70 million years (Jarvis et al., 2014) were exceptionally distant. It was not immediately clear from the paper which laboratory (University of Missouri – St. Louis, University of Leeds) generated the data from these purportedly infected blue‐footed boobies, nor was it obvious at the time that the sequence matched a common parasite that was under study in the same laboratory at the University of Leeds at the same time. Following these first findings, it showed that EMCIR01 was a common parasite of yellow hammers *Emberiza citrinella* in Europe (Dunn et al., 2014) with three closely related (>99% sequence similarity) lineages (EMSPO01, EMRUT01, EMBUC01) in other species of *Emberiza* buntings (Ishtiaq et al., 2007; Nourani et al., 2018; Palinauskas et al., 2013). To explore this remarkable host sharing further, the authors of the Levin et al. (2011) kindly shared samples from four of the infected blue‐footed boobies for planned analyses of nuclear gene sequences along with repeated samples of EMCIR1 isolates from European yellow hammers (Huang et al., 2018). However, when the samples were analysed in MEEL, Lund, they showed to be negative with the standard nested protocol for avian haemosporidians (Hellgren et al., 2004) as well as by a protocol amplifying a conserved region of the rRNA of the parasite's mitochondria (Fallon et al., 2003). It seemed to not be a problem of DNA quality as primers for the host mtDNA (Kocher et al., 1989) amplified strong bands. Why were we unable to obtain parasite sequence from these same samples in Lund? The booby sequences had been generated by one of the coauthors of Levin et al. (2011) at the University of Leeds, UK, in the same laboratory where yellow hammer samples infected by EMCIR01 had been processed in parallel (Dunn et al., 2014). Although not proven, it seems more likely that the records of the *Parahaemoproteus* lineages from boobies in Levin et al. (2011) are a result of cross‐project contamination rather than true biological findings.

The history of erroneous publications of sequence records from avian haemosporidians may be as old as the research field itself. The first published cyt b sequence, thought to be from *Haemoproteus columbae* and obtained from a feral pigeon sampled in Venezuela (Escalante et al., 1998), was later shown to be identical to the lineage GRW02 of the morphological species *Plasmodium ashfordi* (Valkiūnas, et al., 2007). The taxonomic mistake is easy to understand since there were no previous cyt b sequences of *Haemoproteus* parasites, and the result can be explained if this pigeon was simultaneously infected by *H. columbae* and *Plasmodium ashfordi* and that the primers (designed based on *Plasmodium* from mammals) preferentially amplified the *Plasmodium* infection. However, the sequence identity to the lineage GRW02 is puzzling. In 1995, Bensch sent a DNA isolate from a great reed warbler *Acrocephalus arundinaceus* infected with what was thought at the time to be an unknown *Haemoproteus* parasite to the authors for them to use in their study of the phylogeny of mammal malaria parasites. Remarkably, this particular sample was later shown to be infected by GRW02 (Bensch et al., 2000). Over the 20 years that have passed since the publication of GRW02 in a pigeon from Venezuela, this lineage now has plenty of records from Europe and Africa, but has never again been recorded in the Americas or in Columbiform birds. We therefore propose that a more plausible explanation is that the record is a result of either PCR contamination or a mix‐up of samples in the laboratory procedure.

A lineage‐rich cluster of parasites including *Haemoproteus nucleocondensus*, *H. belopolskyi* and *H. payevskyi* are primarily found in warblers of the family Acrocephalidae (Ciloglu et al., 2020; Krizanauskiene et al., 2012; Valkiūnas et al., 2007). The transmission area of the majority of these lineages appear to be restricted to sub‐Saharan Africa, as suggested from the observations that only adult individuals of their afrotropical migratory host species carry infections when sampled in Europe. Most of these lineages appear to be strong host specialists (1–3 hosts) with few records in bird species outside this family of songbirds (Bensch et al., 2009). Notable exceptions to this pattern are reports of the lineages MW1, RW1 and GRW01 in dippers *Cinclus cinclus* (Rojo et al., 2015) and MW1 and RW1 in Iberian shrikes *Lanius meridionalis* (Casanueva et al., 2012; Hernández et al., 2017), both studied in Spain. The isolated findings of these lineages in dippers and grey‐backed shrikes that are both resident species in Spain and distantly related to Acrocephalidae are surprising in the light of the absence of these lineages in many well sampled and more closely related species in the Iberian peninsula (Mata et al., 2015). Twenty‐five of these infected samples were reanalysed in Lund, where primers from other regions of the parasites mtDNA failed amplification. Primers for the hosts amplified strong bands, suggesting that the failure of amplifying the parasites is not explained by degradation of the DNA during the years that passed since the samples were analysed for the original publication. To infer these findings as contamination rather than host shifts and change of transmission area requires a plausible route of how the contamination could have happened. There was a previous study carried out in the same laboratory where the dippers and shrikes were screened, including 149 reed warblers *Acrocephalus scirpaceus* and 39 sedge warblers *Acrocephalus schoenobaenus*, that reported the parasites MW1 and RW1 (Fernández et al., 2010). Hence, we suggest that the findings of the Acrocephalidae parasites in the dippers and shrikes should be interpreted as due to cross‐project contamination.

We have found two more records of these Acrocephalidae‐infecting parasites that also might stem from contamination. A study of house sparrows (*Passer domesticus*) from France reported one bird infected by GRW01 (Bonneaud et al., 2006) which is outside the presumed transmission area (Sub‐Saharan Africa) of this parasite. Contamination might be a more plausible explanation since the study was carried out in the laboratory in Lund where great reed warblers (the main host of GRW01) were screened for parasites at the same time. Unpublished records of MW1 and the similar lineage (COTCOT03) from quails *Coturnix coturnix* in Spain, deposited in MalAvi, are probably a result of contamination from a parallel screening of *Acrocephalus* warblers which was conducted at the same time (A. D. P. Rodríguez, personal communication).

*Plasmodium gallinaceum* is a parasite of the domestic chicken *Gallus gallus domesticus* with a documented transmission area restricted to southern Asia (Valkiūnas, 2005), where it can cause substantial mortality in chicken farms. A finding of the cyt b lineage GALLUS01 (the barcoding marker for *P. gallinaceum*) from passerine birds sampled in Japan (Imura et al., 2011) was therefore surprising, but also worrying since this finding indicated that passerines could carry this parasite from winter quarters in southern Asia to Japan, with potential spread to chicken farms. In the same laboratory as the study by Imura et al. (2011), penguins from a zoo in Japan were recently recorded infected with GALLUS01. This was initially interpreted as *P. gallinaceum* having active transmission in Japan, and these unpublished results were discussed at the 4th International Conference on Malaria and Related Haemosporidian Parasites of Wildlife in Beijing, China (Sehgal, 2019). An alternative explanation, however, was suggested: contamination from the PCR positive control that, in this laboratory, routinely was from an isolate of *P. gallinaceum*. This alternative explanation was supported by reanalyses with primers from elsewhere in the parasite mtDNA genome that failed to amplify haemosporidians from the GALLUS01 positive samples in Imura et al. (2011) or from the zoo penguins.

Although most publications do not specify the sample used for positive controls, many studies have probably used *P. gallinaceum* DNA when screening for haemosporidians in wild birds. This is because *P. gallinaceum* was one of the most well studied avian malaria parasites before the molecular revolution, and multiple isolates of DNA from highly infected chickens have been shared and distributed between laboratories worldwide. If available, using *P. gallinaceum* as a positive control was thus a natural first choice in many laboratories when setting up studies of haemosporidians in wild birds. There are a few reports of GALLUS01 from passerine birds or wild‐caught mosquitoes sampled outside the known transmission area of *P. gallinaceum* (Kim & Tsuda, 2010; Lacorte et al., 2013; Perkins & Schall, 2002). Whether these are true biological findings or results of PCR contamination from positive controls remains unknown but would be worth testing for confirmation.

Common lineages are particularly likely to be the source of contamination because every positive PCR is a potential source. Widespread and generalist parasites are therefore highly vulnerable to this problem, particularly because researchers will not be surprised to find them in new host species or geographic regions. Examples of widespread generalists are the lineages SGS1 and GRW04 of the morphospecies *Plasmodium relictum*, recorded in 132 and 82 host species, respectively (MalAvi database, October 2019). However, pinpointing records, if any, as being due to contamination will remain speculative. A report of GRW04 from one single greylag goose *Anser anser* in Germany (Musa et al., 2018) is indicative of contamination as both the host species and the transmission area are surprising. Also, the lineage GRW04 was amplified in a parallel project carried out in the same laboratory (Musa et al., 2019). Whereas the lineage SGS1 is widespread and common in Eurasia and Africa, there are only two published records from the Americas; in a single tree swallow from Canada (Turcotte et al., 2018) and from several species of hummingbirds and passerines at two sites in Peru (Marzal et al., 2015). Since SGS1‐infected samples had never been analysed in the laboratory recording the infected tree swallow, contamination seems implausible (D. Garant, personal communication). On the contrary, SGS1 is one of the most abundant lineages amplified in the laboratory in Spain where the Peruvian samples were analysed. However, the authors addressed the contamination concerns by stating in their publication that the “molecular analyses were repeated in three independent laboratories to verify the validity and reproducibility of detection of the SGS1 lineage” (Marzal et al., 2015).

## 3 RECOMMENDATIONS AND CONCLUSION

Completely avoiding PCR contamination in publications would require substantial resources and efforts, e.g., by confirming each infection with multiple primer sets, from independent extractions from the same sample or by analyses in different laboratories. However, we can substantially reduce the reporting in publications of erroneous results due to PCR contamination by being open to the possibility that these may happen, even in the best organized laboratories, and reanalyse any samples that produce unexpected or otherwise suspicious results. In Table 1, we provide a checklist of questions to help evaluate whether a finding is the result of contamination; the risk is increasing with the number of these questions that can be answered with a “yes”.

**TABLE 1:** Checklist alerting whether findings of haemosporidian cytochrome b lineages in novel host species may require additional examination before publications to verify that these are not the result of PCR‐contamination. The more of the questions that can be answered by “yes” the more likely that the finding is a result of contamination

Questions	Alert level
Does the host species belong to a different family than previously known host species?	Some
Have microscopic analyses of blood smears failed to find infected cells?	Some
Has the lineage been encountered previously in recent projects in the laboratory?	Medium
Is the main host species of the lineage included in the same experiment?	Medium
If generated by nested PCR, were the setup for the second PCR done at the same workspaces and/or pipettes as the first PCR?	High
Were the PCRs done by beginners in the laboratory, e.g., students?	High
Was the sample run next to another sample shown to be positive for the lineage?	High
Is the lineage the same as in the sample of the positive control?	High
Does the sample fail to amplify with general screening primers?	High

The first measure to take is to reduce the risk of contamination and to monitor background levels of contamination in the laboratory. The detection power of nested PCR comes with a cost; the PCR products from the first PCR can easily contaminate samples in the second PCR. It is therefore strongly advisable to allocate separate locations (preferably separate rooms) and pipettes for DNA extraction, PCR setup and handling of PCR‐products. For monitoring the contamination in the laboratory, the use of negative controls (NTC) should never be compromised. It is important to emphasize that using only one NTC per experiment will mainly test if the reagents in the master mix (including the water) are contaminated. To monitor the presence of low levels of background contamination (e.g., aerosols of previous PCR products that might precipitate in any of the tubes), it is advisable to use multiple NTCs (e.g., 1 out of 8) which will increase the chance of detecting background contamination. If NTCs repeatedly are found positive, determining the lineage of these by sequencing will provide knowledge on which contaminants are present and could possibly help with detecting the source of the contamination.

The way the NTCs are organized in the experiment might also affect the chance of detecting low‐level background contamination. This is illustrated by a study carried out in the laboratory in Lund that investigated 382 samples from birds from the Azores that had overall low rates of infection (Hellgren et al., 2011). All of the NTCs (one out of eight samples) were negative as were most of the samples from 10 species of birds. Five samples from four species, however, were positive for the lineage PARUS1, a common parasite of tits in Europe, but not previously recorded in the species being tested. These samples proved to be blood‐smear negative and, since the analyses of re‐extracted DNA failed amplification, the findings were excluded in the final publication as they were suspected to be due to contamination from a previous project conducted in the same laboratory on blue tits (*Cyanistes caeruleus*) infected with PARUS1 (Stjernman et al., 2008). The contaminated samples were all on the same row of the 96‐plate (row D) whereas the 12 NTC were placed on the bottom (row H). Hence, it was thought that the contamination was from one of the channels in the multipipette (position 4 from the left), which could explain why it did not show up in the NTCs. Arranging the NTCs cross‐wise on the plate (A‐H) would have improved the chances of picking up such a source of contamination.

Many laboratories nowadays use separate protocols for screening (Ciloglu et al., 2019; Fallon et al., 2003) and for the sequencing (Hellgren et al., 2004). Disagreement between repeated PCRs (with the same or different primers) is however expected if infection intensities are low (around the detection limit of the PCR), so it may not be a sign of contamination (Bensch & Hellgren, 2020). However, cases where sequences are obtained from samples that are repeatedly negative by the screening primers should be carefully evaluated. If the lineage is new to the host species (and of a lineage that previously has been amplified in the laboratory), it should be verified by additional investigation. The most convincing test would be to have a subsample of the original sample analysed in a different laboratory; however, that might be difficult to arrange. Because contamination is likely to be from a PCR product of the primers used for sequencing, designing new primers outside this region is a viable alternative. Whole mtDNA genome sequences are available from an increasing number of haemosporidians (Pacheco et al., 2018). Hence, designing primers for novel regions of the mtDNA genome is nowadays quite straightforward.

Samples selected to be used for positive controls are typically from highly infected isolates as one wants a robust sample when checking the success of the assay. However, the repeated use of highly infected samples increases the risk of generating PCR contaminants that may carry over to other samples in the same experiment or form background contamination in the laboratory which could affect later projects. The ignorance of positive controls being the potential source of contamination dominates studies of avian haemosporidians; <5% of the papers included in the MalAvi database have reported the lineage(s) of the sample(s) used as positive controls. Insightful selection of samples for positive controls may also highlight cases of potential contamination. For example, it would be more informative to use samples from a known host‐specific parasite when screening samples from other bird species than using a sample from known generalist parasites. The finding of the former in an unexpected host species would call for checking the sample again, using a new DNA extract or primers from a different region of the parasite's genome. For this reason, *P. gallinaceum* would be an excellent positive control in studies of nongalliform birds.

Collecting blood smears in parallel with blood for molecular analyses is strongly recommended as it allows for confirming the presence of a parasite by microscopic analyses, which effectively removes concerns of contamination. However, PCR‐positive but blood smear negative samples are expected to be relatively frequent due to submicroscopic infection intensities or aborted development of the parasite (Moens et al., 2016). The latter refers to situations where the parasite can infect and replicate in internal organs, but fails to infect blood cells and hence cannot complete transmission as this requires formation of gametocytes in the blood. Therefore, microscopy can verify the presence of a parasite in a sample but if negative, cannot separate PCR contamination from low level or aborted infections.

Finally, mixing up samples in the field or in the laboratory can lead to wrongly assigned parasites to host species. If a tube was mislabelled in the field, analyses of re‐extracted DNA from the original sample will not help. In such cases, it would be informative to examine the host species identity by sequencing a shorter region of the hosts cytochrome b or COI genes that can be amplified by any of many bird‐universal primers (Kocher et al., 1989).

The examples we report here of published records that are possibly the result of PCR contamination have been singled out from cases where we have robust information on the natural occurrence of the parasite (host species and/or geographic region). We expect that these are just the tip of the problem because for most of the >3,900 lineages found to date, we know far too little of haemosporidian natural occurrence to be alerted if a record is improbable. Errors assigning parasites to host species is a problem that goes beyond the study itself, because when entered to common databases, the records will repeat the errors in global analyses of parasite host and distribution ranges (Clark et al., 2014). A relevant question to ask is whether PCR contaminations in publications, and as a consequence falsely assigned host species, have contributed to the general conclusion that haemosporidian parasites mainly evolve by frequent host switching rather than co‐speciation (Fecchio et al., 2018). We think this is unlikely because switching between host species (Ricklefs et al., 2014) and continents (Ellis et al., 2019) have also been inferred from records of closely related but yet different lineages, and because they have different sequences, they cannot be caused by PCR‐contaminations.

We believe that contamination of samples during PCR analysis is not exclusive to haemosporidian research, but an example of a broader problem that applies to molecular identification of other taxonomic groups – notably microorganisms ‐ where opportunities to confirm the findings with alternative methods are restricted. Particularly challenging are metabarcoding studies where identifications are based only on DNA (Bush et al., 2019). For example, contamination, PCR errors and hopping index primers in multiplexed sequence pools will contribute to an omnipresence of low levels of false positives (Ficetola et al., 2015; van der Valk et al., 2020). Repeated analyses of re‐extracted samples combined with careful bioinformatic filtering are hence required steps to minimize the presence of false positives (Ficetola et al., 2015). However, the optimal level of replication is always study‐specific and as for all studies based on PCR amplification of low concentration or degraded DNA, the recommendation to “Look before you leap” (Taberlet et al., 1999) still holds true.

In conclusion, we hope that careful laboratory routines, including following up the presence of amplifications of negative controls, selection of positive controls, and verification of suspicious findings with alternative primers will reduce the publication of contamination records in the future. Accordingly, we encourage reviewers and journal editors to require that unexpected findings should be tested with additional primers before recommending manuscripts for publication.

## AUTHOR CONTRIBUTIONS

S.B. conceived the study and wrote the article. All authors read and commented on the draft manuscript and approved final version.
